# Static Postural Balance in Modern Pentathletes: A Pilot Study

**DOI:** 10.3390/ijerph16101760

**Published:** 2019-05-18

**Authors:** Dorota Sadowska, Tomasz Sacewicz, Małgorzata Lichota, Justyna Krzepota, Maria Ładyga

**Affiliations:** 1 Department of Physiology, Institute of Sport—National Research Institute in Warsaw, 01-982 Warsaw, Poland; maria.ladyga@insp.waw.pl; 2 Department of Biomechanics and Computer Science, Faculty of Physical Education and Sport in Biała Podlaska, Józef Piłsudski University of Physical Education in Warsaw, 21-500 Biała Podlaska, Poland; tomasz.sacewicz@awf-bp.edu.pl; 3 Department of Posture Correction and Compensation, Faculty of Physical Education and Sport in Biała Podlaska, Józef Piłsudski University of Physical Education in Warsaw, 21-500 Biała Podlaska, Poland; malgorzata.lichota@awf-bp.edu.pl; 4 Department of Physical Culture and Health Promotion, University of Szczecin, 71-065 Szczecin, Poland; justyna.krzepota@usz.edu.pl

**Keywords:** static postural balance, modern pentathletes, shooting position

## Abstract

Postural balance is a key element of shooting effectiveness, which determines the outcome of modern pentathlon competitions. The aim of the study is to examine the postural balance of 27 pentathletes (12 females and 15 males; mean age: 18.0 ± 1.8 years), and 26 physically active, untrained subjects (12 females and 14 males; mean age: 22.5 ± 1.4 years), and to investigate the impact of footwear on the stability of the shooting position in pentathletes. *Methods*: Static postural balance was examined during quiet stance in four test conditions (standing in footwear with eyes opened, standing in footwear with eyes closed, standing barefoot with eyes opened, and standing barefoot with eyes closed). During each postural balance measurement, the participant remained still on the platform, with their arms in front of their body. Postural balance in the shooting position was only evaluated in the group of pentathletes. The athlete was asked to assume a comfortable shooting position on the platform and to aim at the target. Standard pentathlon targets and pistols were used in the study. Measurements were carried out twice (barefoot and in footwear). *Results*: In all conditions, pentathletes achieved lower values of posturographic measures than in the control group. In non-visual conditions, measures describing the surface area of the centre of pressure decreased in pentathletes and increased in the control group. Both pentathletes and non-athletes were equally stable barefoot as in footwear. Footwear did not affect postural sway in the shooting position in pentathletes. *Conclusions*: Pentathletes were found to have significantly better stance stability and were less vision-dependent in postural balance than untrained subjects. Bearing in mind that the shooting position of pentathletes was as stable barefoot as in footwear, the main factors which were most likely responsible for minimising body oscillations in the pentathletes were their high level of concentration and conscious control of body alignment.

## 1. Introduction

The modern pentathlon combines five different disciplines: fencing, swimming, horseback riding, and laser run, which is running and shooting combined. Due to the very diverse nature of these disciplines, the modern pentathlon requires comprehensive physical fitness and is considered to be extremely difficult. Particularly high requirements related to postural balance control are imposed by fencing and shooting. Both disciplines develop strong postural balance, but each of them has a different effect on postural balance characteristics. Fencing is a dynamic sport which causes continuous destabilisation and thus particularly develops dynamic balance control. Shooting, however, is a static activity that requires strict control of body posture. The task of pistol shooters is to maintain a maximally stable bipedal posture in order to stabilize the weapon [1]. Important reports on the postural balance of fencers and pistol shooters were provided by Herpin et al. [1]. Regardless of visual information (eyes opened or eyes closed), pistol shooters achieved significantly better results in postural balance measurements without sensory conflict (when the support surface was fixed), while fencers demonstrated better balance with sensory conflict (eyes closed, with sway-referenced support surface).

Sensory information about posture from the somatosensory, visual, and vestibular systems is crucial for postural balance control [2,3]. It has been reported that for most people (including athletes), the absence of visual feedback input impairs postural balance inter alia [4,5]. Some studies have shown that the proportions of the three sensory systems involved in postural balance control differ in non-athletes and athletes [1,6], in athletes at various competition levels [6,7], as well as between athletes practising different kinds of sports [1,8,9]. There are sport activities, such as dance, in which strong visual dependency has been observed [10,11] as well as disciplines in which vestibular and proprioceptive information play special roles in postural balance control, for example judo [9] or shooting [1,12]. According to Herpin et al. [1], pistol shooters cannot use visual information preferentially to control postural stability, as they use their vision mainly for sighting. In comparative studies of postural balance involving experienced pistol shooters and non-athletes, it was indeed observed that whereas both visual input and foot position contributed to stance stability in controls, the stability of shooters was determined mainly by foot position [12].

Despite the fact that pistol shooting is part of the modern pentathlon, the postural balance of pentathletes and sports shooters may differ significantly. Unfortunately, there are no reports in the literature on the postural balance of pentathletes. Pentathletes undergo completely different training than shooters because they have to simultaneously master five disciplines at a very high level. Moreover, the requirements imposed by the laser run are very different from those of pistol shooting. Unlike sport shooters, pentathletes need to aim at the target while being in a state of physical exhaustion, after covering a certain running distance. Fatigue induced by physical effort is an additional factor that destabilises the shooting position, which has a negative impact on the stability of the hold [13,14].

Footwear, and the type of footwear, have a significant impact on postural balance due to the influence of the quality of sensory feedback from the feet [15,16]. In recent years, numerous studies have been carried out on the influence of various types of footwear on postural balance, mainly in the context of preventing injuries among athletes [17,18] as well as improving postural balance and preventing falls in the elderly inter alia [15,19,20]. It has been reported that appropriately selected, comfortable footwear does not impair postural balance [21] and perhaps even supports a stabilising posture [22].

Considering the key role of postural balance in pistol shooting and the many hours of training spent in footwear, which is an important tool in athletic training, it seems important to verify whether footwear has a positive impact on the postural balance of pentathletes, and in particular, whether it has a stabilising effect on their shooting position. Moreover, in light of the lack of reports concerning postural balance and the role of visual information in postural balance control in pentathletes, the aims of our research to assess postural balance during bipedal upright stance among pentathletes and non-athlete controls, including an analysis of the impacts of vision and footwear on postural balance control, as well the assessment of the stability of the shooting position and the impact exerted by footwear in pentathletes.

## 2. Materials and Methods

### 2.1. Participants

The study involved 27 pentathletes (12 females and 15 males; mean age: 18.0 ± 1.8 years), who were members of the Polish Association of Modern Pentathlon. All athletes were competing at the national, or both the national and international, levels. The average body height and mass of the pentathletes were 178.0 ± 8.4 cm and 66.5 ± 8.2 kg, respectively.

The control group was composed of 26 physically active physical education students (12 females and 14 males) who did not have any previous experience in modern pentathlon or shooting and did not practise sports professionally. The mean age, body height, and body mass of the participants were 22.5 ± 1.4 years, 174.0 ± 10.5 cm and 70.5 ± 9.2 kg, respectively.

Prior to the study, each participant was provided with detailed information about the test procedures and the research methodology. Each participant was examined individually, but all postural balance measurements were made during the same test session. Written informed consent was sought from the participants or their legal guardians in the case of underage subjects. The protocol of the study conformed to the recommendations of the Declaration of Helsinki and was approved by the Research Ethics Committee at the Institute of Sport—National Research Institute in Warsaw (decision no. KEBN-18-41-MŁ).

### 2.2. Procedure

Postural balance was examined using the Zebris FDM-2 Force Distribution Measuring System (Zebris Medical Gmbh, Isny, Germany), which also records the centre of pressure (COP) signal. The Zebris platform includes individually-calibrated motion detectors which make it possible to analyse the density distribution of static and dynamic forces acting on the ground while standing and walking. As each subject stood on the platform (dimensions: 212 × 60.5 × 2.1 cm; number of miniature force sensors: 15,360), the force exerted by their feet was recorded by the sensors at a sampling rate of 120 Hz.

Postural balance during quiet stance was evaluated under four conditions: (1) standing in footwear with eyes opened; (2) standing in footwear with eyes closed; (3) standing barefoot with eyes opened and (4) standing barefoot with eyes closed. The completion order of the four tasks was randomised for all participants. At the command of the investigator, the participant stood on the platform. During each postural balance measurement, the participant remained still on the platform, with their arms in front of their body. After each completed measurement, the participant left the platform. The platform was calibrated before each measurement.

Postural balance in the shooting position was evaluated only in the group of pentathletes. The athlete was asked to assume a comfortable standing shooting position on the platform and to aim at the target after hearing the command. Standard pentathlon targets and pistols were used in the study. Measurements were carried out twice (barefoot and in footwear).

Each postural balance measurement lasted 40 seconds. The first and last five seconds of each recording were removed and the 30 s records of COP displacements were further analysed. The coordinates of the instantaneous COP were calculated with WinFDM Stance processing software (Zebris Medical Gmbh, Isny, Germany) (Table 1).

### 2.3. Statistical Analysis

Statistical analyses were conducted using STATISTICA 13.0 software (Dell inc., Tulsa, OK, USA) [23]. The normality of the distribution of the analysed variables was verified using the Shapiro-Wilk test. Due to the fact that the distribution of all variables differed from the normal distribution, a Box-Cox transformation was used to normalise the variables [24]. The data were analysed using a three-factor ANOVA (2 × 2 × 2), and the eta-squared (η^2^) effect size was calculated to analyse the interaction effects. The within-subject factors were vision (eyes opened, eyes closed) and footwear (in footwear, barefoot), and the between-subject factor was the group (pentathletes, control). Post-hoc Bonferroni tests were applied. One-way ANOVA (factor: footwear) was used to evaluate the effect of footwear on the stability of the pentathletes’ shooting positions. The accepted level of statistical significance was *p* ≤ 0.05.

## 3. Results

A three-factor ANOVA did not show statistically significant interactions between the group, vision and footwear factors. The interactions between the vision and footwear factors were also insignificant.

For the sway path length of the COP (SP) and the average velocity of the COP (V), the level of statistical significance was recorded for the group, vision and footwear factors (Table 2). Athletes were characterised by significantly lower values of these parameters than in the control group (group effect for SP: F = 4.77; *p* = 0.0302; group effect for V: F = 4.59; *p* = 0.0333). In the no-vision conditions, an increase in parameters was observed in both groups (vision effect for SP: F = 54.61; *p* < 0.0001; vision effect for V: F = 54.82; *p* < 0.0001). Moreover, in both groups, higher values of parameters were observed in the measurements for footwear (footwear effect for SP: F = 5.04; *p* < 0.0258; footwear effect for V: F = 5.11; *p* = 0.0248). The mean values of measures describing COP shifts recorded in the four conditions in pentathletes and non-athletes are presented in Table 3.

**Figure 1:**
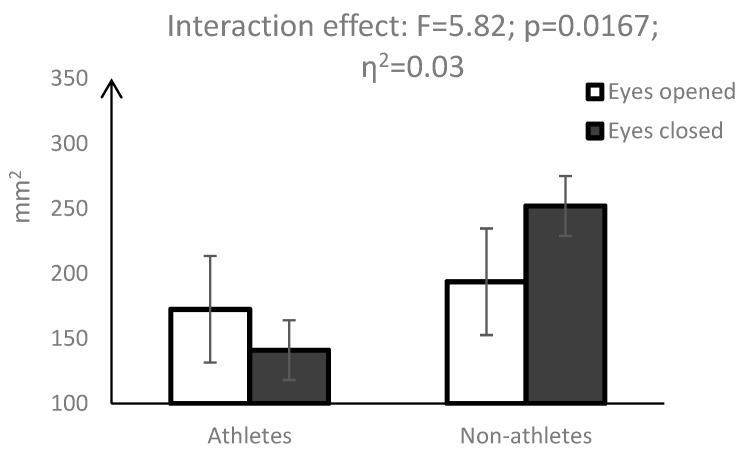
Mean values and 95% confidence interval for the ellipse of the COP shifts area (AoE) along with the results of the interaction between group and vision factors.

**Figure 2:**
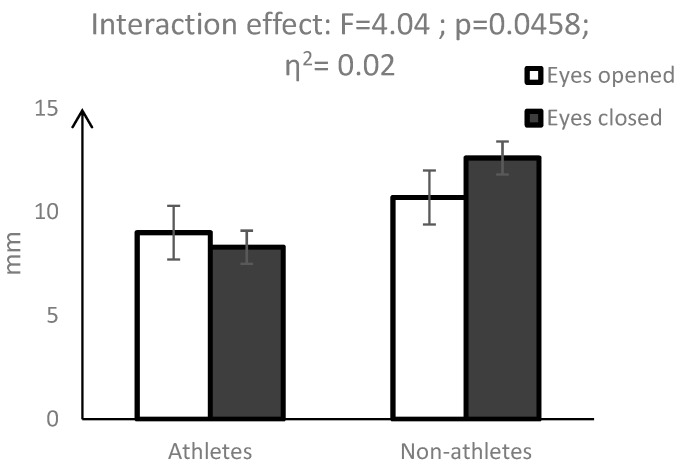
Mean values and 95% confidence interval for the width of the COP ellipse (WoE), along with the results of the interaction between group and vision factors.

**Figure 3:**
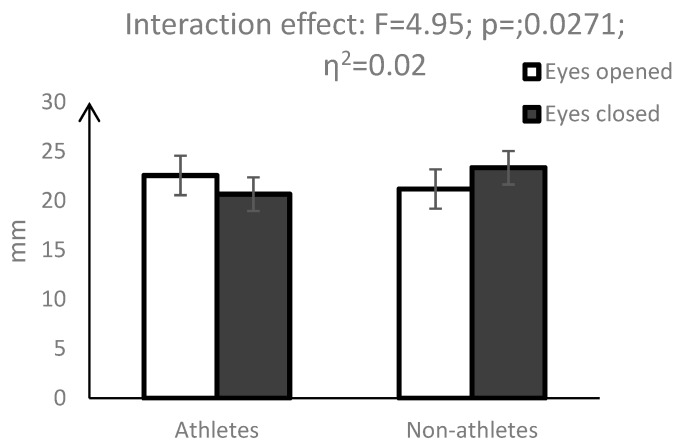
Mean values and 95% confidence interval for the height of the COP ellipse (HoE), along with the results of the interaction between group and vision factors.

A statistically significant interaction effect (vision × group) was observed for area of the centres of pressure (AoE) (F = 5.82, *p* = 0.0167) (Figure 1), width of the ellipse (WoE) (F = 4.04; *p* = 0.0458) (Figure 2) and height of the ellipse (HoE) (F = 4.95; *p* = 0.0271) (Figure 3). While eye closure caused a significant decrease in the above parameters in the group of pentathletes, the opposite effect was observed in the control group.

For AoE and WoE, ANOVA showed a statistically significant group effect (F = 15.78, *p* = 0.0008; and F = 45.46, *p* = 0.0000, respectively). Irrespective of the measurement conditions (eyes opened, eyes closed), pentathletes were characterised by significantly lower values in comparison to the control group (Table 2). In the case of HoE, the footwear effect was statistically significant (F = 7.56; *p* = 0.0065). The mean values of measures describing the surface area of the COP recorded under four conditions in pentathletes and non-athletes are presented in Table 3.

The assessment of the impact of footwear on the stability of the shooting position of pentathletes did not reveal statistically significant differences on any of the parameters analysed (Table 4).

## 4. Discussion

The study showed that a quiet standing posture was more stable among pentathletes compared to the control group. Our observations are consistent with the results of comparative studies of postural balance in shooters inter alia [12,25] and archers [26]. However, no similar studies have been conducted among pentathletes.

Aalto et al. [25] observed that rifle shooters and pistol shooters were characterised by lower COP velocities than non-athletes in a standing position, both with opened and closed eyes. Su et al. [12] also noted significantly lower values of mean and maximum COP velocity in shooters compared to the control group. Our research had similar findings. Regardless of the measurement conditions (in footwear/barefoot; eyes opened/eyes closed), pentathletes were characterised by significantly lower values of the posturographic measures analysed than non-training participants. We are convinced that the differences observed are the result of the training undertaken by the pentathletes. Success in shooting sports is determined by a high level of postural balance, good coordination of the segments of the body and high concentration at the time of the execution of the shot [1,26]. That is why the main element of the shooting training of pentathletes, similar to shooters, is learning to maintain a stable upright bipedal posture for a long time, in order to stabilise the gun. In both pentathletes and the control group, eye closure caused a significant increase in the COP shifts (SP and V). The results obtained confirmed previous observations conducted among various groups of athletes [8,27].

Significant interactions (vision × group) of measures describing the surface area of the COP also indicated different changes in the postural balance of pentathletes and non-athletes taking place in the non-visual condition. While for non-athletes, closing the eyes caused a significant increase in AoE, WoE and HoE, which indicated a deterioration of postural balance, for pentathletes, these indices were reduced. Similar results were obtained by Su et al. [12] and Aalto et al. [25]; however, their observations concerned COP velocity and the dispersion index. In the study of Su et al. [12], the lack of visual information did not disturb the postural balance of shooters, but negatively affected the postural balance of non-training participants. Similarly, in the study of Aalto [25], the non-visual condition significantly reduced body sway velocity in shooters as compared with the control subjects. Our results thus indicate that the postural balance of pentathletes as well as of shooters is less reliant on visual control. During shooting, vision is focused on targeting and cannot be used to control postural balance [1,25]. We suppose that a smaller contribution of visual control to the postural balance of pentathletes is compensated for by a much larger share of proprioceptive and vestibular control. Previous studies demonstrated that due to the requirements of each sport, athletes use specific sensory information to organise posture [8,28].

As far as the impact of footwear is concerned, both groups examined obtained significantly lower values of SP, V and HoE in barefoot than in footwear measurements. Numerous reports indicate that the type of footwear, as well as its modifications (e.g., the use of shoe insoles), affect postural balance by means of stimulating the tactile as well as proprioceptive systems [15,16,29]. An analysis of postural balance in men wearing different types of footwear revealed no differences between standard shoe and barefoot conditions; however, wearing sandals significantly increased postural sway [29]. On the other hand, in a study conducted on a group of older women, Lord et al. [22] observed the highest level of static balance for bare feet or low-heeled shoes and the worst balance for high-heeled shoes.

We also found that in the case of SP and V, the differences in the average values of measures recorded in footwear and barefoot were significantly higher in the pentathletes than in the control group. This observation could additionally confirm the stronger dependence of pentathletes on proprioceptive control, but the lack of statistical significance, probably caused by the large dispersion of results, does not allow such conclusions to be drawn.

Based on the results of research showing that proprioceptive information is the main source of feedback in the process of postural balance control in shooters, the next goal of our research was to compare the stability of the shooting position of pentathletes in two different conditions, namely in footwear and barefoot. The study conducted on air pistol athletes by Hawkins et al. [30] suggests that stance width affects postural and pistol stability. Of the five successive foot positions (heels 30, 45, 60, 75, and 90 cm apart), the first one (30 cm stance width) allowed the athletes to minimise COP velocity and COP path length to the greatest extent. In turn, Aalto et al. [25] reported that the supportive clothing (which also includes special footwear) used by shooters during competitions reduces the sway velocity both in visual and non-visual conditions.

Our findings showed that, contrary to quiet standing, the pentathletes’ shooting position was as steady in footwear as it was barefoot. Regardless of whether the athletes took a shooting position in comfortable sports shoes or barefoot, the body sway was very similar. A standing shooting position is very mentally and physically demanding and is incomparably more difficult than a natural standing position. That is why experienced rifle shooters have been found to have less body sway than novice control groups [31]. A regression analysis conducted by Ball et al. [32] indicated that body sway is related to performance and aim point fluctuation in rifle shooters. When body sway increased, performance decreased and aim point fluctuation increased. We believe that the observed lack of differences is due to high concentration as well as conscious control and minimisation of body oscillation by pentathletes. These processes play crucial roles in the reduction of postural sway in the shooting position.

## 5. Conclusions

To conclude, our data showed that pentathletes had significantly better stance stability than untrained controls. While measures describing the surface area of the COP in pentathletes decreased in a non-visual condition, they increased in the control group. The different postural balance changes in the two groups caused by eye closure indicate a smaller contribution of visual information to postural balance control in pentathletes. Both pentathletes and non-training persons were more stable barefoot than in footwear. Wearing sport shoes, compared to being barefoot, did not impact the stability of the shooting position in pentathletes. Maintaining a stable shooting position is a difficult task that requires high concentration as well as conscious control and minimisation of body oscillation. These processes probably play a key role in the reducing postural sway and stabilising the shooting position in pentathletes.

## Figures and Tables

**Table 1:** Specification of posturographic measures analysed. COP = centre of pressure.

Parameter	Description of Parameter
COP shifts	
SP [mm]	Sway path length of COP
V [mm/s]	Average velocity of COP
COP surface area	
AoE [mm^2^]	Area of centre of pressure (calculated from COP shifts in such a way that 95% of data are within ellipsoid and 5% are outside of it)
WoE [mm]	Width of ellipse (length of ellipse in medial-lateral direction)
HoE [mm]	Height of ellipse (length of ellipse in anterior-posterior direction)

**Table 2:** Effects of the between-subject factor (group: pentathletes/controls) and two within-subject factors (vision: eyes opened, eyes closed; footwear: in footwear, barefoot) and interaction of the between-subject factor with the within-subject factors on posturographic measures.

Parameter	Group	Vision	Footwear	Group x Vision	Group x Footwear
COP shifts					
SP [mm]	F = 4.77	F = 54.61	F = 5.04	F = 2.87	F = 2.58
	*p* = 0.0302 *	*p* < 0.0001 ***	*p* = 0.0258 *	*p* = 0.0917 ^NS^	*p* = 0.1096 ^NS^
	η^2^ = 0.02	η^2^ = 0.21	η^2^ = 0.02	η^2^ = 0.01	η^2^ = 0.01
V[mm/s]	F = 4.59	F = 54.82	F = 5.11	F = 2.94	F = 2.61
	*p* = 0.0333 *	*p* < 0.0001 ***	*p* = 0.0248 *	*p* = 0.0877 ^NS^	*p* = 0.1096 ^NS^
	η^2^ = 0.02	η^2^ = 0.25	η^2^ = 0.02	η^2^ = 0.01	η^2^ = 0.01
COP surface area					
AoE [mm^2^]	F = 15.78	F = 0.84	F = 2.46	F = 5.82	F = 2.04
	*p* < 0.0001 ***	*p* = 0.3605 ^NS^	*p* = 0.1180 ^NS^	*p* = 0.0167 *	*p* = 0.1551 ^NS^
	η^2^ = 0.07	η^2^ = 0.00	η^2^ = 0.01	η^2^ = 0.03	η^2^ = 0.01
WoE [mm]	F = 45.46	F = 0.86	F = 0.00	F = 4.04	F = 1.33
	*p* < 0.0001 ***	*p* = 0.3545 ^NS^	*p* = 0.9944 ^NS^	*p* = 0.0458 *	*p* = 0.2501 ^NS^
	η^2^ = 0.18	η^2^ = 0.00	η^2^ = 0.00	η^2^ = 0.02	η^2^ = 0.01
HoE [mm]	F = 0.00	F = 0.56	F = 7.56	F = 4.95	F = 1.33
	*p* = 0.973 ^NS^	*p* = 0.4542 ^NS^	*p* = 0.0065 **	*p* = 0.0271 *	*p* = 0.2501 ^NS^
	η^2^ = 0.00	η^2^ = 0.00	η^2^ = 0.04	η^2^ = 0.02	η^2^ = 0.01

SP = sway path length of the COP; V = average velocity of the COP; AoE = ellipse of the COP shifts area; WoE = width of the COP ellipse; HoE = height of the COP ellipse; * = *p* ≤ 0.05; ** = *p* < 0.01; *** = *p* < 0.001; ^NS^ = no statistical significance.

**Table 3:** Means and standard deviations for posturographic measures recorded under four conditions in pentathletes and non-athletes.

Parameter	Group	Open Eyes M ± SD	Closed Eyes M ± SD	In Footwear M ± SD	Barefoot M ± SD
COP shifts					
SP (mm)	Athletes	177.3 ± 52.3	231.4 ± 66.5	222.1 ± 64.8	186.7 ± 61.7
	Control	184.5 ± 61.2	299.8 ± 138.2	248.3 ± 127.8	236.0 ± 114.9
V (mm/s)	Athletes	5.9 ± 1.7	7.7 ± 2.2	7.4 ± 2.1	6.2 ± 2.0
	Control	6.1 ± 2.0	9.9 ± 4.6	8.2 ± 4.2	7.8 ± 3.8
COP surface area					
AoE (mm^2^)	Athletes	172.5 ± 150.1	141.1 ± 82.5	175.3 ± 137.1	138.4 ± 101.8
	Control	193.7 ± 164.7	252.0 ± 200.4	233.1 ± 214.1	212.7 ± 151.6
WoE (mm)	Athletes	9.0 ± 4.6	8.3 ± 3.1	8.8 ± 3.8	8.5 ± 4.1
	Control	10.7 ± 3.2	12.6 ± 4.3	11.4 ± 4.0	11.9 ± 3.8
HoE (mm)	Athletes	22.6 ± 7.5	20.7 ± 6.6	23.5 ± 6.9	19.7 ± 6.9
	Control	21.2 ± 11.7	23.4 ± 9.2	23.5 ± 12.6	21.1 ± 8.0

SP = sway path length of the COP; V = average velocity of the COP; AoE = ellipse of the COP shifts area; WoE = width of the COP ellipse; HoE = height of the COP ellipse.

**Table 4:** Means and standard deviations for posturographic measures recorded among pentathletes during aiming-in-shooting position, with and without footwear; along with the results of one-way ANOVA with footwear factor.

Parameter	In Footwear	Barefoot	ANOVA		
	M ± SD	M ± SD	F	P	η^2^
COP shifts					
SP [mm]	282.6 ± 177.1	234.2 ± 146.9	1.49	0.2285	0.03
V [mm/s]	9.4 ± 5.9	7.8 ± 4.9	1.50	0.2257	0.03
COP surface area					
AoE [mm^2^]	150.8 ± 75.8	148.9 ± 89.1	0.06	0.8154	0.01
WoE [mm]	8.8 ± 3.2	9.2 ± 3.4	0.05	0.8242	0.01
HoE [mm]	21.8 ± 7.2	19.9 ± 6.2	0.61	0.4381	0.01

SP = sway path length of the COP; V = average velocity of the COP; AoE = ellipse of the COP shifts area; WoE = width of the COP ellipse; HoE = height of the COP ellipse.
